# Adverse Events After Carbon-Ion Radiotherapy (CIRT) for Hepatocellular Carcinoma and Risk Factors for Biliary Stricture After CIRT: A Retrospective Study

**DOI:** 10.3390/cancers17152542

**Published:** 2025-07-31

**Authors:** Keita Maki, Hiroaki Haga, Tomohiro Katsumi, Kyoko Hoshikawa, Fumiya Suzuki, Fumi Uchiyama, Takashi Kaneko, Masashi Koto, Yoshiyuki Ueno

**Affiliations:** 1 Department of Gastroenterology, Yamagata University Faculty of Medicine, Yamagata 990-9585, Japan; k-maki@med.id.yamagata-u.ac.jp (K.M.); t-katsumi@med.id.yamagata-u.ac.jp (T.K.); to.kyoko@med.id.yamagata-u.ac.jp (K.H.); f-suzuki@med.id.yamagata-u.ac.jp (F.S.); fuchiyama@med.id.yamagata-u.ac.jp (F.U.); y-ueno@med.id.yamagata-u.ac.jp (Y.U.); 2 Department of Radiology, Division of Radiation Oncology, Yamagata University Faculty of Medicine, Yamagata 990-9585, Japan; takashi.kaneko@med.id.yamagata-u.ac.jp (T.K.); koto.masashi@med.id.yamagata-u.ac.jp (M.K.)

**Keywords:** adverse events after carbon-ion radiotherapy, biliary stricture, carbon-ion radiotherapy, hepatocellular carcinoma

## Abstract

**Background/Objectives**: This study investigated the timing of adverse events (AEs) after carbon-ion radiotherapy (CIRT) for hepatocellular carcinoma (HCC) and identified the risk factors for biliary stricture post CIRT. **Methods**: This retrospective study included 103 patients with HCC who had undergone CIRT (60 Gy/4 fractions). The onset, frequency, and grade of AEs after CIRT were analyzed. HCC was classified into perihilar and distal types to assess the frequency of biliary stricture, and the risk factors for biliary stricture were investigated. **Results**: AEs after CIRT were more frequent in patients with liver dysfunction, skin redness/dermatitis, and pigmentation. Biliary stricture occurred long after CIRT (3.0–17.0 months). Most AEs were of grade 1–2. Grade ≥ 3 AEs included biliary stricture (2.9%) and radiation gastric ulcer (1.0%), whereas grade 5 AEs included biliary stricture (1.9%). Biliary stricture was exclusively observed in patients with perihilar-type HCC. Among patients with perihilar-type HCC, those having a tumor in the portal vein trunk branch area were more prone to biliary stricture than those with a tumor in the primary portal vein branch area (*p* = 0.0018), and all grade ≥ 3 biliary strictures (2.9%) were observed in the portal vein trunk branch area. Patients with perihilar-type HCC and biliary stricture were more likely to have macrovascular invasion (*p* = 0.0052) and previous local therapy targeting the perihilar region (*p* = 0.0371) than those without biliary stricture. **Conclusions**: This study reported the detailed data of AEs post CIRT for HCC and the risk factors for biliary stricture post CIRT.

## Simple Summary

This study investigated the timing of adverse events (AEs) after carbon-ion radiotherapy (CIRT) for hepatocellular carcinoma (HCC) and the risk factors for biliary stricture associated with high-grade AEs. We retrospectively analyzed 103 patients who received CIRT (60 Gy/4 fractions). The timing of most AEs was relatively easy to identify; however, biliary stricture was observed over a wide range and required careful attention. The potential risk factors for biliary stricture after CIRT include perihilar-type HCC, HCC involving the portal vein trunk branch area, presence of macrovascular invasion, and history of treatment in the perihilar area. These results may help improve patient management and follow-up strategies after CIRT.

## 1. Introduction

Hepatocellular carcinoma (HCC) is the most frequent type of liver cancer and a leading cause of cancer-related deaths worldwide [1,2,3,4]. In Japan, since 2022, particle therapy for HCC of a size of 4 cm or larger that is difficult to surgically remove has been included under insurance coverage, expanding treatment options for liver cancer. Particle therapies, such as carbon-ion radiotherapy (CIRT) and proton beam radiotherapy, have gained attention in recent years due to their high efficacy and safety. CIRT offers higher linear energy transfer and better radiobiological effectiveness than proton beam therapy and X-ray therapy [5,6,7,8,9]. The Bragg peak enables precise dose delivery to the tumor while minimizing exposure to surrounding healthy tissue [10,11,12,13,14]. In addition, CIRT requires fewer radiation sessions, thereby reducing the treatment burden [10,15,16].

Although CIRT has shown a high local control rate, adverse events (AEs) following irradiation may occur. However, systematic studies detailing the onset and severity of AEs after CIRT are lacking.

Among the AEs observed after CIRT, biliary stricture can lead to recurrent obstructive jaundice and cholangitis, posing challenges in management. We have experienced several cases in which biliary stricture occurred after CIRT, leading to repeated cholangitis and a poor prognosis. Therefore, we tried to investigate the risk factors associated with the occurrence of biliary stricture following CIRT. Although AEs following CIRT for HCC have been examined [17], risk factors associated with biliary strictures occurring after CIRT have not been much investigated. This study focused on the time to onset and severity of AEs occurring after CIRT and analyzed risk factors for the serious AEs of biliary stricture.

## 2. Materials and Methods

### 2.1. Patient Population

This study included 103 patients who had undergone CIRT for HCC at our hospital from November 2022 to August 2024 and were monitored for over 6 months. The diagnosis of HCC, vascular invasion, lymph node involvement, and distant metastasis was assessed using contrast-enhanced computed tomography (CT) and/or magnetic resonance imaging (MRI). In cases where imaging results were inconclusive for HCC, histological confirmation was obtained via tumor biopsy. None of the included patients were lost to follow-up.

### 2.2. CIRT

The spot scanning method and a rotating-gantry beam system, allowing irradiation from 360°, were employed. Before treatment planning, fiducial metallic markers were implanted near the tumor to ensure precise positioning. To maintain target reproducibility, a low-temperature thermoplastic sheet, vacuum bag, and respiratory-gated irradiation system were used for CT-based radiotherapy planning. Gross tumor volume (GTV) was determined using dynamic contrast-enhanced CT and/or MRI, while clinical target volume included a 7 mm margin from the gross tumor volume. Treatment planning was conducted using a CT-based three-dimensional system with RayStation (RaySearch Laboratories, Stockholm, Sweden) that was optimized using robustness tools. Set-up errors were compensated for with a 2 mm margin in left–right, anterior–posterior, and superior–inferior directions. A software setting of 2% was used for the range uncertainty parameter.

For treatment planning, CT scan was taken using 4D-CT linked to respiratory movement. Then, CT images were created with 10 phases in 10% increments from 0 to 100%, with the inhalation phase at 0% and the exhalation phase at 50%. Subsequently, contouring was performed on the 50% exhalation phase image, and the internal target volume was set by combining the CT images with the maximum phase range where the GTV movement was within 3 mm, and only the phases within that range were used for actual irradiation.

Orthogonal fluoroscopy and radiography were used to confirm and correct the radiation field before each treatment. The prescribed dose of CIRT was expressed in Gy and calculated as the absorbed dose multiplied by the relative biological effectiveness of carbon ions. According to the institutional protocol for HCC, 60 Gy/4 fractions was recommended. Four-fraction treatment was administered four times a week for 4 consecutive days. Regarding the constraints for organs at risk, we set the remnant liver volume to be more than 500 cc for doses less than 30 Gy for the liver parenchyma. We set the D2 cc to be less than 30 Gy for the digestive tract.

### 2.3. Clinical Outcome Evaluation

Patients were assessed at 1 month post CIRT and then every 3 months for the first 2 years, every 6 months for the next 2 years, and annually thereafter. Follow-up assessments included physical examinations, laboratory tests, and dynamic contrast-enhanced CT or MRI scans. The final follow-up was conducted in March 2025.

Toxicity related to CIRT was assessed using the Common Terminology Criteria for Adverse Events (CTCAE) version 5.0. CTCAE uses a grading system from 1 to 5 to classify the severity of AEs, where 1 indicates mild, 2 indicates moderate, 3 indicates severe, 4 indicates life-threatening, and 5 indicates death. Tumor progression and symptoms attributed to the tumor or other treatments for recurrent tumors were not considered in the assessment of CIRT-related toxicity. Assessments included physical examinations (skin condition, digestive and respiratory symptoms, pleural and abdominal effusion, and jaundice), blood tests (total bilirubin [mg/dL], albumin [g/dL], aspartate aminotransferase [U/L], alanine aminotransferase [U/L], platelet count [×10^3^/µL], prothrombin time, prothrombin time-international normalized ratio, alpha-fetoprotein [ng/mL], and protein-induced vitamin K absence or antagonist-II [mAU/mL]), and imaging tests (contrast-enhanced CT or MRI) at 3, 6, 9, 12, 15, 18, 21, and 24 months post treatment. AEs occurring within 90 days of CIRT initiation were defined as early AEs, whereas those occurring after 90 days were defined as late AEs. Biliary stricture was defined as the new onset of bile duct dilatation on CT or MRI following CIRT, with no evidence of such dilatation on pretreatment imaging. Cases with bile duct dilatation attributable to tumor infiltration, either before or after CIRT, were excluded. Biliary stricture requiring therapeutic intervention was defined as a case in which obstructive jaundice and cholangitis were suspected because of biliary dilatation accompanied by elevated hepatic and biliary enzyme levels, fever, and upper abdominal pain.

For CIRT, we defined local recurrence as radiographic findings of tumor enlargement or new early enhanced areas within the treatment field, in which contrast enhancement disappeared after CIRT, based on the RECIST v1.1 guidelines. The overall survival (OS) rate was calculated from the initial treatment date of CIRT to the date of death from any cause. The local control (LC) rate was calculated from the initial treatment date of CIRT to the date of local recurrence. To determine the effect of CIRT, OS and LC rates were measured at 1 and 2 years.

To ensure consistency and objectivity in imaging assessment, the diagnosis of biliary stricture was independently confirmed by a panel of four experts, comprising two hepatologists and two radiologists from our institution.

### 2.4. Definitions of Perihilar-Type HCC and Distal-Type HCC Based on Their Proximity to the Portal Vein Trunk or Primary Branch

Perihilar-type HCC is defined as a tumor located within 1 cm of the portal vein trunk or primary branch, whereas distal-type HCC refers to a tumor located 1 cm or more away from these structures (Supplementary Figure S1). Furthermore, HCC that is perihilar-type and within 1 cm of the portal vein trunk is classified as perihilar-type HCC in the portal vein trunk branch area, whereas HCC located 1 cm or more away from the portal vein trunk is categorized as perihilar-type HCC in the primary portal vein branch area (Supplementary Figure S1).

### 2.5. Statistical Analysis

Patients with perihilar-type HCC and primary portal vein branch involvement and those with portal vein trunk branch involvement as well as patients with perihilar-type HCC with and without biliary stricture following CIRT were compared using the t-test for continuous variables and the χ^2^ test or Fisher’s exact test for categorical variables. Due to the small sample size, the Shapiro–Wilk test was conducted prior to the *t*-test to confirm data normality. OS and LC rates were analyzed using the Kaplan–Meier method. Statistical analyses were conducted using GraphPad Prism-v9, with a significance threshold set at *p* < 0.05.

Propensity score matching (1:2) was performed with a caliper width of 0.2 using the MatchIt package (version 4.5.5) in R (version 4.4.2). After matching, the groups were compared using *t*-test for continuous variables and Fisher’s exact test for categorical variables.

### 2.6. Ethics Approval

This retrospective study was approved by the Ethics Review Committee of Yamagata University School of Medicine (Approval No. 2023-110).

## 3. Results

### 3.1. Patient Characteristics in the CIRT Group (Table 1)

The study included 79 men and 24 women with a median age of 76 years (range: 47–97 years) who had undergone CIRT. The etiologies of liver disease included hepatitis B (*n* = 19), hepatitis C (*n* = 25), non-B/non-C hepatitis (*n* = 19), alcoholic hepatitis (*n* = 18), metabolic dysfunction-associated steatohepatitis (*n* = 18), autoimmune hepatitis (*n* = 1), and primary biliary cholangitis (*n* = 3). The modified albumin–bilirubin (mALBI) grades were distributed as follows: grade 1 (*n* = 50), grade 2a (*n* = 24), grade 2b (*n* = 26), and grade 3 (*n* = 3), with a median ALBI score of −2.603 (range: from −3.668 to −1.049). Based on the Barcelona Clinic Liver Cancer (BCLC) staging system, 63 patients were classified as having stage A, 21 as stage B, and 19 as stage C (Table 1).

Among the 103 patients, 48 received CIRT as their initial treatment, whereas 55 had undergone prior treatments, including surgery (*n* = 16), radiofrequency ablation (*n* = 20), transarterial chemoembolization (TACE, *n* = 29), stereotactic radiation therapy (SRT, *n* = 3), and systemic therapy (atezolizumab + bevacizumab, *n* = 14; lenvatinib, *n* = 8). All three cases in which SRT was performed, CIRT was administered at a location separate from the SRT field to avoid high cumulative doses to the bile duct. The median maximum tumor diameter was 54.5 mm (range: 12–142 mm). Most patients (*n* = 83) had a single liver tumor, whereas 20 patients had two tumors. Two tumors were in close proximity to each other and were irradiated as a single tumor. Microvascular invasion was absent in 84 patients and present in 19 patients (Table 1).

### 3.2. AEs After CIRT

**Figure 1:**
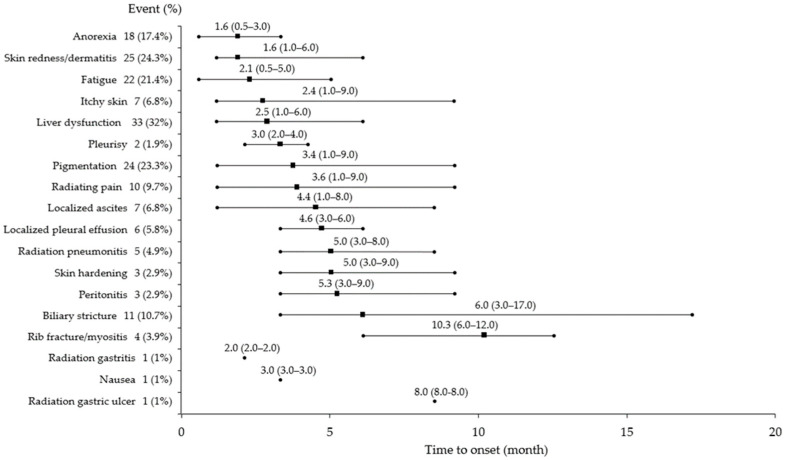
Onset of adverse events following CIRT in patients with HCC. CIRT, carbon-ion radiotherapy; HCC, hepatocellular carcinoma.

The median follow-up period was 17.8 months (range: 6.2–27.4 months). All 103 patients completed the treatment course successfully without any interruptions. Figure 1 illustrates the incidence and onset of AEs observed during the follow-up period.

**Figure 2:**
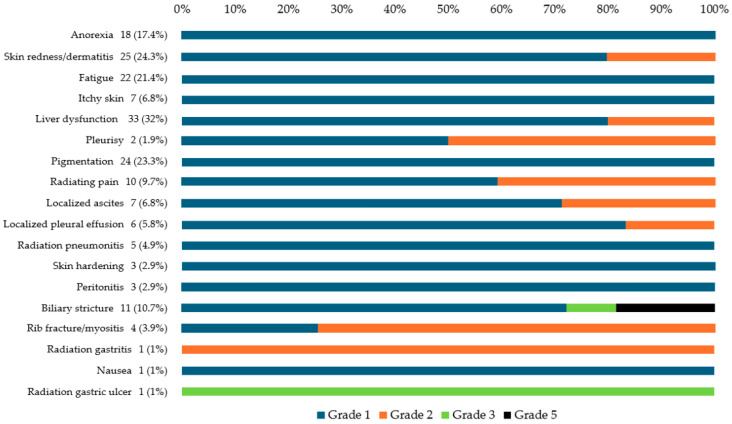
Severity of adverse events following CIRT in patients with HCC. CIRT, carbon-ion radiotherapy; HCC, hepatocellular carcinoma.

The most common AEs were liver dysfunction (*n* = 33, 32%), skin redness/dermatitis (*n* = 25, 24.3%), pigmentation (*n* = 24, 23.3%), fatigue (*n* = 22, 21.4%), and anorexia (*n* = 18, 17.4%). Early AEs (onset within 3 months) included anorexia (median: 1.6 months), skin redness/dermatitis (median: 1.6 months), fatigue (median: 2.1 months), itchy skin (median: 2.4 months), and liver dysfunction (median: 2.5 months). Late AEs (onset after 3 months) were biliary stricture (median: 6.0 months) and rib fracture/rib myositis (median: 10.3 months). Figure 2 illustrates the grading of AEs observed during the follow-up period, with most events classified as grade 1 or 2. Transient increases in liver dysfunction were observed, followed by rapid decreases. Anorexia, fatigue, and nausea did not necessitate intervention. Skin redness/dermatitis was managed conservatively with no intervention or by applying steroid ointment. Itchy skin, pigmentation, and skin hardening did not require intervention. Pleurisy was managed conservatively or with nonsteroidal anti-inflammatory drugs. Peritonitis no longer required intervention. Localized pleural effusion and ascites were managed conservatively or with diuretics. Rib fracture/rib myositis and radiating pain were managed conservatively or with nonsteroidal anti-inflammatory drugs. Radiation pneumonitis did not require oxygen in any patient and was monitored. Radiation gastritis was managed conservatively with peristaltic stimulants.

Grade 3 or higher AEs were rare, occurring in only four patients (3.9%): three with biliary stricture and one with a radiation-induced gastric ulcer. The patients with biliary stricture underwent endoscopic retrograde cholangiopancreatography and stenting; however, two patients died of uncontrolled cholangitis. The patient with gastric ulcer was managed with oral antacids and red blood cell transfusion, leading to improvement (Figure 2).

### 3.3. Characteristics of Patients with Perihilar-Type HCC and Distal-Type HCC Receiving CIRT (Table 2)

Among the 103 patients treated with heavy ion radiotherapy, 64 had perihilar-type HCC and 39 had distal-type HCC. When the total number of intrahepatic tumors was two, the tumors were closely adjacent in all cases. A tumor was classified as periportal HCC if it was located within 1 cm of the portal vein trunk or a primary branch.

Patients with perihilar-type HCC showed a higher proportion of BCLC stage C (*p* = 0.0095) and macrovascular invasion (MVI, *p* = 0.0078) than those with distal-type HCC. In addition, patients with perihilar-type HCC had a larger tumor diameter than those with distal-type HCC (median size: 63.0, range: 11.5–93 years; *p* = 0.0095) and were more likely to have a biliary stricture (*p* = 0.0061).

### 3.4. Comparison of Patients with Perihilar-Type HCC with and Without Biliary Stricture (Table 3)

**Figure 3:**
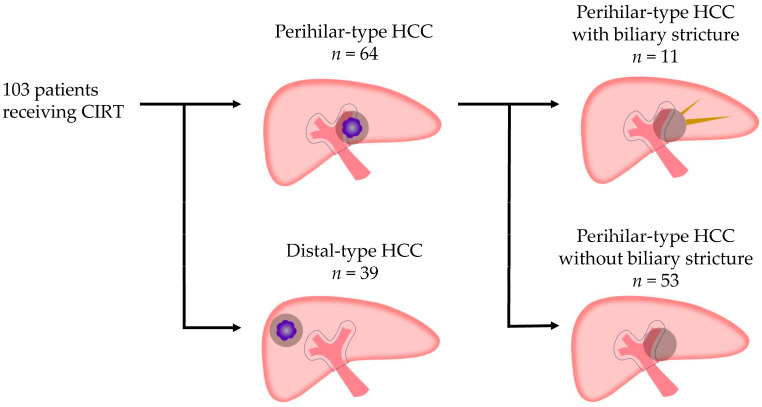
Flowchart of selection of patients with perihilar-type HCC and biliary stricture. HCC, hepatocellular carcinoma.

Patients with perihilar-type HCC (*n* = 64) were classified into those with (*n* = 11) and without (*n* = 53) biliary stricture (Figure 3). Of the 11 patients with biliary stricture, 8 had grade 1 changes without symptoms or abnormalities in blood tests. In these 8 cases, only mild biliary dilatation was observed on follow-up CT, and no therapeutic intervention was required. The remaining 3 patients with grade ≥3 biliary stricture had tumors located in the portal vein trunk branch area and developed cholangitis. None of the patients who developed biliary stricture exhibited tumor recurrence in the perihilar region. When the total number of intrahepatic tumors was two, if any tumor was located within 1 cm of the portal vein trunk, it was classified as perihilar-type HCC in the portal vein trunk branch area.

Patients with perihilar-type HCC with biliary stricture exhibited a higher incidence of BCLC stage C (*p* = 0.0247) and MVI (*p* = 0.0052) than those without biliary stricture. Among patients with perihilar-type HCC and biliary stricture, MVI was present in 63.6% (5 Vp3, 1 Vp2, 1 Vv2), whereas among those with perihilar-type HCC without biliary stricture, MVI was observed in 18.9% (5 Vp3, 3 Vp2, 1 Vv3, 1 Vv2). Patients with perihilar-type HCC and biliary stricture also had a higher rate of previous local therapy targeting the perihilar region than those without biliary stricture (*p* = 0.0371). In particular, 45.5% of patients with perihilar-type HCC with biliary stricture had a history of treatment in the perihilar area (three cases of TACE and two cases of SRT for portal vein tumor thrombus), whereas only 15.1% of those without biliary stricture had undergone a previous local therapy targeting the perihilar region (seven cases of TACE and one case of SRT). Furthermore, patients with perihilar-type HCC and biliary stricture had a higher incidence of tumors in the portal vein trunk branch area than those without biliary stricture (*p* = 0.0018). Biliary stricture was present in 33.3% of patients with perihilar-type HCC in the portal vein trunk branch area and in 2.9% of those with perihilar-type HCC in the primary portal vein branch area. In three patients with grade 3 or higher biliary stricture, perihilar-type HCC was present in the portal vein trunk branch area. Two of these three patients had a history of SRT for portal vein tumor thrombosis in the perihilar area.

Univariate analysis revealed potential risk factors for biliary stricture. To reduce the influence of confounding variables, propensity score matching (1:2) was performed. Matching was conducted between patients with (*n* = 11) and without (*n* = 22) biliary stricture based on age, sex, and ALBI score. The results indicated that patients with perihilar-type HCC with biliary stricture had a higher incidence of tumors in the portal vein trunk branch area than those without biliary stricture (*p* = 0.0024) (Supplementary Table S1).

### 3.5. OS and LC Rates After CIRT

**Figure 4:**
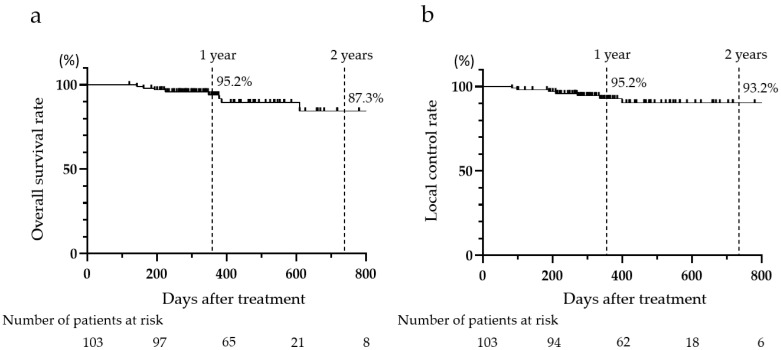
Overall survival rate and local control rate of all patients after carbon-ion radiotherapy. (**a**) overall survival rate, (**b**) local control rate.

The 1- and 2-year OS rates were 92.5% and 87.3%, and the 1- and 2-year LC rates were 92.5% and 93.2%, respectively. During the follow-up period, nine patients died due to disease progression (*n* = 5), cholangitis (*n* = 2), lung cancer (*n* = 1), and heart failure (*n* = 1) (Figure 4).

## 4. Discussion

This study reports the onset, frequency, and grade of AEs after CIRT for HCC and the risk factors for biliary stricture post CIRT. Patients were followed up for approximately 1.5 years after CIRT, and the AEs that occurred during that period were analyzed in detail. In addition, HCC was classified into perihilar and distal types to assess the frequency of biliary stricture, with the perihilar type further categorized into portal vein trunk branch area and primary portal vein branch area to investigate the risk factors for biliary stricture.

The study findings indicated detailed onset timing of AEs post CIRT. AEs of grade 3 or higher included biliary stricture (2.9%) and radiation gastric ulcer (1.0%). Grade 5 AEs included biliary stricture (1.9%). Biliary stricture was observed in patients with perihilar-type HCC but not in those with distal-type HCC. Among patients with perihilar-type HCC, the incidence of biliary stricture was higher in the portal vein trunk branch area than in the primary portal vein branch area (33.3% vs. 2.9%, *p* = 0.0018). Among patients with perihilar-type HCC with biliary stricture, the proportion of those with previous local therapy targeting the perihilar region (*p* = 0.0371) and MVI (*p* = 0.0052) were higher than those without biliary stricture. HCC involving the portal vein trunk branch area, presence of MVI, and a history of treatment in the perihilar area were identified as potential risk factors for biliary stricture post CIRT.

CIRT is recommended for patients with HCC of 4 cm or more in size who are not amenable to conventional treatments and have Child–Pugh A or B liver function. It is a viable option for patients who are ineligible for surgical resection. The dose and fractionation schedule for CIRT may vary among institutions, but at our center, we typically administer 60 Gy in four fractions. Given the surgical risks associated with advanced age, CIRT is increasingly favored over hepatectomy.

In our study, most AEs occurred within 6 months of CIRT, with late AEs, such as biliary stricture (median: 6.0 months) and rib fracture/myositis (median: 10.3 months), appearing later. Moreover, the onset of biliary stricture varied from 3.0 to 17.0 months.

Most AEs were of grade 1–2, and CIRT was typically well-tolerated by elderly patients and those with comorbidities. Meanwhile, grade ≥3 AEs, including biliary stricture and a radiation-induced gastric ulcer were observed in three and one patient, respectively. Biliary stricture was observed in patients with perihilar-type HCC but not in those with distal-type HCC. Moreover, in patients with perihilar-type HCC, the incidence of biliary stricture was higher in the portal vein trunk branch area than in the primary portal vein branch area (33.3% vs. 2.9%). CIRT for perihilar-type HCC in the portal vein trunk branch area may cause bile duct stenosis, potentially leading to grade 5 AEs. Therefore, performing long-term monitoring of bile duct strictures is crucial after CIRT, and the location of HCC in the portal vein trunk branch area is a risk factor for biliary stricture.

Although there have been reports of biliary strictures as AEs for radiation therapy and proton beam therapy in HCC [18,19], no clinical studies have directly compared biliary stricture after CIRT, proton beam radiotherapy, and SRT in patients with HCC with similar tumor and background characteristics. Regarding post-RT biliary strictures, Eriguchi et al. reported 4.0% [20] and Yu et al. reported that 24% of patients developed biliary strictures, with 4% requiring intervention [18]. Zhang et al. reported that the incidence rate of bile duct stenosis after proton beam therapy was 2.2% (2 out of 90 patients; 1 patient had grade 3–4 and one patient had grade 5) [17], and in all cases, the HCC was located in the hilum of the liver. Currently, there are no established values regarding the incidence or severity of biliary stricture following treatments such as CIRT, proton beam radiotherapy and SRT, and the current understanding is based primarily on individual reports from medical institutions.

Radiation-induced damage triggers inflammation and promotes the transformation of fibroblasts into myofibroblasts [21,22,23], leading to excessive production of collagen and other extracellular matrix components and thereby resulting in radiation-induced fibrosis [21,24,25]. In a previous clinical study, biliary stenosis was reported after SBRT for HCC in adjacent areas of the liver. This suggests that excessive radiation may be a risk factor [20]. Similar findings have been reported in a previous study on large animals [26].

Once biliary stricture occurs, managing cholestasis becomes challenging due to radiation-induced fibrosis. While bile duct toxicity after SRT [20,27,28] has been studied, there is limited research on bile duct toxicity after CIRT. In this study, biliary strictures were observed in patients with perihilar-type HCC but not in those with distal-type HCC. Furthermore, among patients with perihilar-type HCC, the frequency of biliary strictures was higher in the portal vein trunk branch area than in the primary portal vein branch area (33.3% vs. 2.9%, *p* = 0.0018). This difference may be attributed to the increased radiation dose near the hepatic hilum as the tumor approached the portal vein trunk. Patients with perihilar-type HCC and biliary stricture had a higher incidence of previous local therapy targeting the perihilar region (*p* = 0.0371) and more cases of MVI (*p* = 0.0052). There were also cases in which TACE or SRT was performed in perihilar-type HCC before CIRT, but CIRT was selected due to difficulty in LC. Inflammation induced by TACE and SRT treatments in the hepatic hilum may contribute to fibrosis around the bile duct.

To mitigate the effects of confounding factors, we performed 1:2 propensity score matching. Perihilar-type HCC cases with biliary stricture had a significantly higher frequency of tumors located in the portal vein trunk branch region than those without biliary stricture (*p* = 0.0024). Nonetheless, because of the limited number of cases, the findings should be interpreted with caution.

Cases of biliary stricture following local treatment for HCC have been reported previously. RFA can cause biliary stricture because of RFA-related thermal injuries [29], and TACE can cause biliary stricture following biliary cellular apoptosis and/or necrosis resulting from hypoxia through occlusion of macrovessels and/or microvessels [30]. This suggests that pretreatment before CIRT may affect biliary stricture. Of the 11 cases of biliary stricture in this study, 8 were of grade 1, asymptomatic, and showed no abnormalities in blood tests. In these eight cases, only mild biliary dilatation was observed on follow-up CT, without any clinical symptoms or laboratory abnormalities, and no intervention was required. All three patients of grade ≥3 biliary stricture had HCC in the portal vein trunk branch area and developed cholangitis. In three patients, one of whom underwent endoscopic biliary stent placement, cholangitis improved and is currently under observation. Two patients died because of a failure to control cholangitis despite endoscopic biliary stent placement. The first patient developed bile duct stricture 5 months after CIRT and died 11 months following CIRT. The second patient developed bile duct stricture 10 months after CIRT and died 19 months following CIRT.

Regarding the outcomes of CIRT, a previous prospective study by Shibuya et al. reported that the 2-year OS rate was 82.8% and the 2-year LC rate was 92.6% [31], whereas in the present study, the 2-year OS rate was 87.3% and the 2-year LC rate was 93.2%, indicating no significant difference compared with previous reports.

This study had some limitations, including its single-center retrospective design, 60 Gy/4 fractions for obtaining results, and potential patient selection bias. Regarding multivariate analysis of risk factors for biliary stricture, statistical reliability was low because of the extremely low events per variable (EPV). Therefore, the risk factors for biliary stricture must be interpreted considering that they are based on univariate findings. In the future, accumulating a larger number of cases and stratifying data to adjust for confounding factors will be necessary. In this study, MVI was assessed in a binary manner (positive/negative), without considering the extent or degree of vascular invasion (e.g., Vp classification). Therefore, the potential impact of MVI severity on the stratification of bile duct stricture risk was not determined. Future studies incorporating detailed classifications of MVI may enable a more nuanced risk assessment.

For future investigations, we intend to consider more direct insights into the etiology of this complication with dosimetric analysis of the radiation dose to the bile duct. Future research should develop strategies to reduce treatment toxicity, such as dose/fractionation adjustments or alternative modalities.

This study reported the detailed onset time of AEs post CIRT for HCC and identified risk factors for biliary stricture post CIRT. Biliary stricture, occurring in the long term after irradiation, necessitates a sufficient follow-up period. Biliary stricture was observed in patients with perihilar-type HCC, and HCC involving the portal vein trunk branch area, presence of MVI, and a history of treatment in the perihilar area were identified as risk factors for biliary stricture post CIRT.

## 5. Conclusions

This study provides insights into the onset time of AEs for HCC and risk factors for biliary stricture post CIRT. It is a valuable resource for managing AEs and holds significance for future CIRT studies. Further research with increased case numbers is anticipated.

## Figures and Tables

**Table 1:** Characteristics of patients receiving carbon-ion radiotherapy (CIRT).

	Carbon-Ion Radiotherapy (*n* = 103)
Age (year)	Median: 76 (range: 47–97)
Sex (male/female) (%)	79 (76.7)/24 (23.3)
Etiology (HBV/HCV/non-B, non-C/alcohol/NASH/AIH/PBC) (%)	19 (18.4)/25 (24.3)/19 (18.4)/18 (17.5)/18 (17.5)/1 (1)/3 (2.9)
mALBI grade (1/2a/2b/3) (%)	50 (48.5)/24 (23.3)/26 (25.2)/3 (2.9)
ALBI score	−2.603 (−3.668 to −1.049)
BCLC stage (A/B/C) (%)	63 (61.2)/21 (20.4)/19 (18.4)
First treatment/pretreatment (surgery/RFA/TACE/SRT/systemic therapy) (%)	48 (46.6)/55 (53.4)
Size (mm)	Median: 54.5 (range: 12–142)
Total number of intrahepatic tumors (1/2) (%)	83 (80.6)/20 (19.4)
MVI (−/+) (%)	84 (81.6)/19 (18.4)
Blood biochemistry, median (range)	
T-bil level (mg/dL)	0.70 (0.40–2.7)
Alb level (g/dL)	4.0 (2.5–5.1)
AST level (U/L)	31.0 (12–222)
ALT level (U/L)	26.0 (5.0–135)
PLT count (10^3^/µL)	151 (39–382)
PT%	94.0 (40–123)
PT-INR	1.05 (0.87–1.95)
AFP level (ng/mL)	8.3 (2.0–20,000)
PIVKA-II level (mAU/mL)	232.0 (6.7–81,886)

**Table 2:** Characteristics of patients with perihilar-type and distal-type hepatocellular carcinoma (HCC) treated with carbon-ion radiotherapy (CIRT).

	Perihilar-Type HCC (*n* = 64)	Distal-Type HCC (*n* = 39)	*p*-Value
Age (year), median (range)	75 (47–91)	77 (49–97)	0.7652
Sex (male/female) (%)	47 (73.4)/17 (26.6)	32 (82.1)/7 (17.9)	0.3481
Etiology (HBV/HCV/non-B, non-C/alcohol/NASH/AIH/PBC) (%)	11 (17.2)/14 (21.9)/14 (21.9)/ 10 (15.6)/12 (18.7)/1 (1.6)/2 (3.1)	8 (20.5)/11 (28.2)/5 (12.8)/8 (20.5)/ 6 (15.4)/0 (0)/1 (2.6)	0.8859
mALBI grade (1/2a/2b/3) (%)	30 (46.9)/12 (18.7)/21 (32.8/1 (1.6)	20 (51.3)/12 (30.8)/5 (12.8)/2 (5.1)	0.0652
ALBI score	−2.586 (−3.415 to −1.143)	−2.586 (−3.415 to −1.143)	0.4496
BCLC stage (A/B/C) (%)	36 (56.2)/10 (15.6)/18 (28.1)	27 (69.2)/10 (25.6)/2 (5.1)	0.0095
First treatment/pretreatment (surgery/RFA/TACE/SRT/systemic therapy) (%)	28 (43.8)/36 (56.2)	20 (51.3)/19 (48.7)	0.5425
Size (mm), median (range)	63.0 (11.5–142)	46.0 (15–130)	0.0059
Total number of intrahepatic tumors (1/2) (%)	50 (78.1)/14 (21.9)	33 (84.6)/6 (15.4)	0.4555
MVI (−/+) (%)	47 (73.4)/17 (26.6)	37 (94.9)/2 (5.1)	0.0078
Biliary stricture (−/+) (%)	53 (82.8)/11 (17.2)	39 (100)/0 (0)	0.0061
Blood biochemistry, median (range)			
T-bil level (mg/dL)	0.80 (0.40–2.7)	0.75 (0.40–2.5)	0.6754
Alb level (g/dL)	4.0 (2.5–4.9)	3.9 (2.5–5.1)	0.3298
AST level (U/L)	36.0 (13–222)	26.0 (12–83)	0.0676
ALT level (U/L)	28.0 (8.0–135)	24.0 (5–95)	0.2279
PLT count (10^3^/µL)	150 (39–346)	155 (55–382)	0.6248
PT%	94.0 (41–123)	93.0 (40–112)	0.6165
PT-INR	1.04 (0.87–1.95)	1.05 (0.93–1.90)	0.7841
AFP level (ng/mL)	14.6 (2.0–20,000)	4.9 (2.0–20,000)	0.2621
PIVKA-II level (mAU/mL)	217.0 (6.7–51,166)	288.0 (12.0–81,886)	0.9624

**Table 3:** Characteristics of patients with perihilar-type hepatocellular carcinoma (HCC) with and without biliary stricture treated with carbon-ion radiotherapy (CIRT).

	Perihilar-Type HCC with Biliary Stricture (+) (*n* = 11)	Perihilar-Type HCC Without Biliary Stricture (−) (*n* = 53)	*p*-Value
Age (year), median (range)	75 (66–90)	76 (47–91)	0.9786
Sex (male/female) (%)	9 (81.8)/2 (18.2)	38 (71.7)/15 (28.3)	0.7124
Etiology (HBV/HCV/non-B, non-C/alcohol/NASH/AIH/PBC) (%)	2 (18.2)/3 (27.3)/4 (36.4)/0 (0)/ 2 (18.2)/0 (0)/0 (0)	9 (17.0)/11 (20.8)/10 (18.8)/10 (18.8)/10 (18.8)/1 (1.9)/2 (3.8)	0.6755
mALBI grade (1/2a/2b/3) (%)	6 (54.5)/1 (9.1)/4 (36.4)/0 (0)	24 (45.3)/11 (20.8)/17 (32.1)/1 (1.9)	0.8542
ALBI score	−2.652 (−3.415 to −1.984)	−2.586 (−3.019 to −1.143)	0.3662
BCLC stage (A/B/C) (%)	4 (36.4)/0 (0)/7 (63.6)	32 (60.4)/9 (17.0)/12 (22.6)	0.0247
First treatment/pretreatment (surgery/RFA/TACE/SRT/systemic therapy) (%)	4 (36.4)/7 (63.6)	24 (45.3)/29 (54.7)	0.7426
Previous local therapy targeting the perihilar region (−/+) (%)	6 (54.5)/5 (45.5)	45 (84.9)/8 (15.1)	0.0371
Size (mm), median (range)	64.0 (42–110)	60.0 (12–142)	0.7551
Total number of intrahepatic tumors (1/2) (%)	10 (90.9)/1 (9.1)	40 (75.5)/13 (24.5)	0.4306
MVI (−/+) (%)	4 (36.4)/7 (63.6)	43 (81.1)/10 (18.8)	0.0052
Location (primary portal vein branch area/portal vein trunk branch area) (%)	1 (9.1)/10 (90.9)	33 (62.3)/20 (37.7)	0.0018
Blood biochemistry, median (range)			
T-bil level (mg/dL)	0.80 (0.50–2.5)	0.70 (0.40–2.7)	0.7140
Alb level (g/dL)	4.0 (3.3–4.9)	3.9 (2.5–4.4)	0.2574
AST level (U/L)	33.5 (13–67)	36.0 (14–222)	0.4833
ALT level (U/L)	29.5 (10–70)	28.0 (8–135)	0.6963
PLT count (10^3^/µL)	148 (59–291)	151 (39–346)	0.6665
PT%	100 (65–111)	94.0 (41–123)	0.5041
PT-INR	1.00 (0.94–1.35)	1.04 (0.87–1.95)	0.5359
AFP level (ng/mL)	4.2 (2.0–20,000)	18.2 (2.0–20,000)	0.8742
PIVKA-II level (mAU/mL)	270.5 (29–14,678)	150.0 (6.7–51,166)	0.4299

## Data Availability

The data supporting the findings of this study are available from the corresponding author (H.H.) upon reasonable request.

## Supplementary Materials

The following supporting information can be downloaded at: https://www.mdpi.com/article/10.3390/cancers17152542/s1, Figure S1: Classification of perihilar-type and distal-type hepatocellular carcinoma (HCC), with further subdivision of perihilar-type HCC; Table S1: Characteristics of perihilar-type hepatocellular carcinoma (HCC) patients with and without biliary stricture treated with carbon-ion radiotherapy (CIRT) after propensity score matching (1:2).

## Abbreviations

The following abbreviations are used in this manuscript:

AEs	adverse events
ABLI score	albumin–bilirubin grade
BLCL	Barcelona Clinic for Liver Cancer
CIRT	carbon-ion radiotherapy
CT	computed tomography
CTCAE	Common Terminology Criteria for Adverse Events
EPV	events per variable
GTV	gross tumor volume
HCC	hepatocellular carcinoma
LC	local control
mALBI	modified albumin–bilirubin
MRI	magnetic resonance imaging
MVI	macrovascular invasion
OS	overall survival
SRT	stereotactic radiation therapy
TACE	transarterial chemoembolization
