# Supplementary material for: Adverse Events After Carbon-Ion Radiotherapy (CIRT) for Hepatocellular Carcinoma and Risk Factors for Biliary Stricture After CIRT: A Retrospective Study
Source: Cancers (Basel). 2025 Jul 31;17(15):2542. doi: 10.3390/cancers17152542 (PMC12346405; doi:10.3390/cancers17152542)
Supplement: Supplementary file 1 [file cancers-17-02542-s001.zip › Cancers Suplementary figure s1.pptx]

## Slide 1
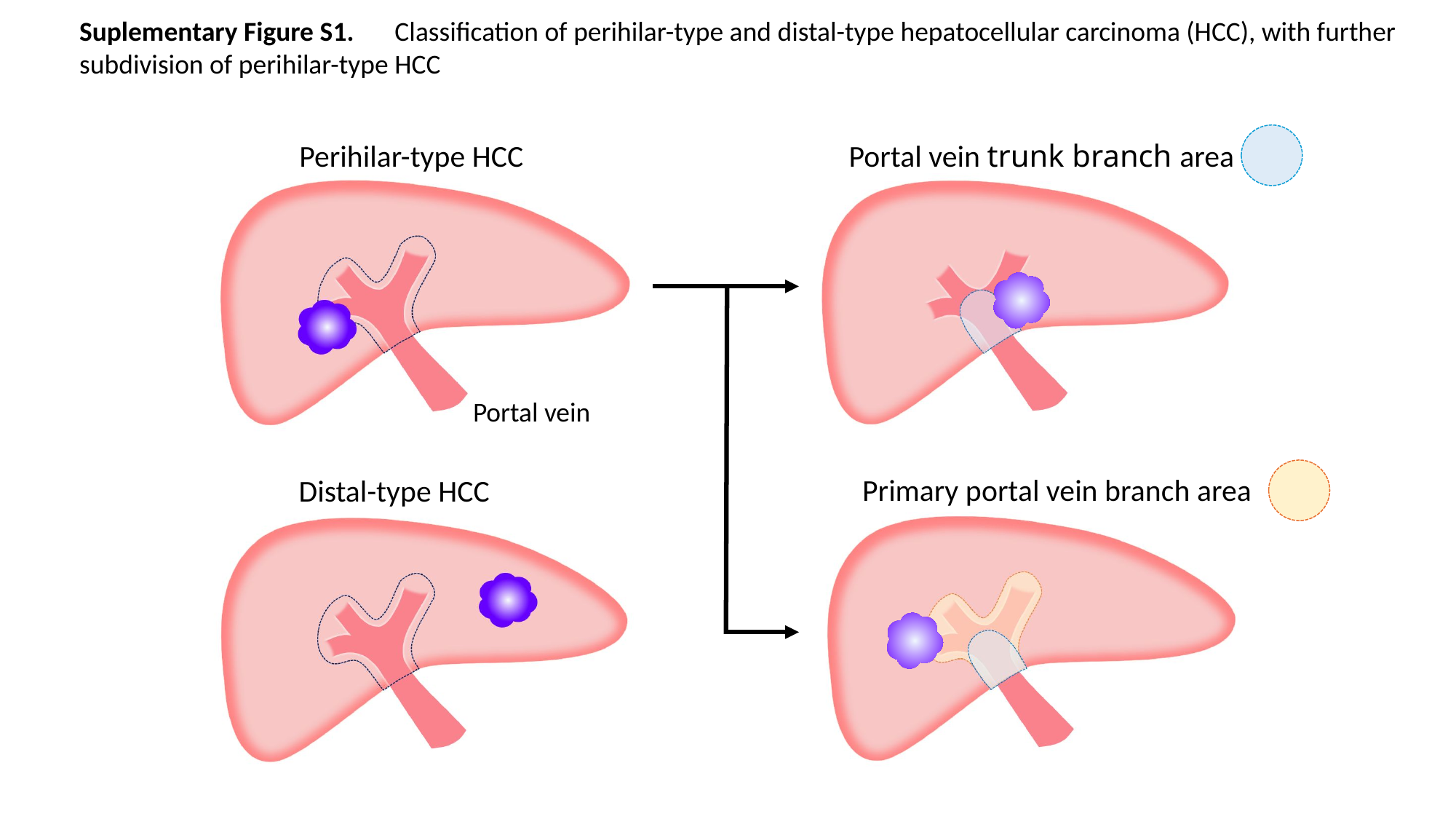

Suplementary Figure S1.　Classification of perihilar-type and distal-type hepatocellular carcinoma (HCC), with further subdivision of perihilar-type HCC
Perihilar-type HCC
Portal vein trunk branch area
Portal vein
Primary portal vein branch area
Distal-type HCC
