# Supplementary material for: Adverse Events After Carbon-Ion Radiotherapy (CIRT) for Hepatocellular Carcinoma and Risk Factors for Biliary Stricture After CIRT: A Retrospective Study
Source: Cancers (Basel). 2025 Jul 31;17(15):2542. doi: 10.3390/cancers17152542 (PMC12346405; doi:10.3390/cancers17152542)
Supplement: Supplementary file 1 [file cancers-17-02542-s001.zip › Cancer Supplementary Table s1.pdf]

Supplementary Table S1. Characteristics of perihilar-type hepatocellular carcinoma (HCC) patients with and without biliary stricture treated with carbon-ion radiotherapy (CIRT) after propensity score matching (1:2).

	**Perihilar-Type HCC with Biliary Stricture (+) (*n* = 11)**	**Perihilar-Type HCC Without Biliary Stricture (−) (*n* = 22)**	***p* Value**
Age (year), median (range)	75 (66–90)	73.5 (56–89)	0.3223
Gender (male/female) (%)	9 (81.8)/2 (18.2)	22 (100)/0 (0)	0.1042
ALBI score	−2.652 (−3.415 to −1.984)	−2.586 (−2.988 to −1.414)	0.9383
BCLC stage (A/B/C) (%)	4 (36.4)/0 (0)/7 (63.6)	15 ()/2 ()/5 ()	0.0723
Previous local therapy targeting the perihilar region (−/+) (%)	6 (54.5)/5 (45.5)	17 ()/5 ()	0.2400
Size (mm), median (range)	64.0 (42–110)	61.5 (20–123)	0.6736
MVI (−/+) (%)	4 (36.4)/7 (63.6)	17 ()/5 ()	0.0518
Location (primary portal vein branch area/portal vein trunk branch area) (%)	1 (9.1)/10 (90.9)	15 ()/7 ()	0.0024
